# Prediction accuracies for growth and wood attributes of interior spruce in space using genotyping-by-sequencing

**DOI:** 10.1186/s12864-015-1597-y

**Published:** 2015-05-09

**Authors:** Omnia Gamal El-Dien, Blaise Ratcliffe, Jaroslav Klápště, Charles Chen, Ilga Porth, Yousry A El-Kassaby

**Affiliations:** Department of Forest and Conservation Sciences, Faculty of Forestry, The University of British Columbia, 2424 Main Mall, Vancouver, British Columbia V6T 1Z4 Canada; Department of Genetics and Physiology of Forest Trees, Faculty of Forestry and Wood Sciences, Czech University of Life Sciences Prague, Kamycka 129, 165 21 Prague 6, Czech Republic; Department of Biochemistry and Molecular Biology, Oklahoma State University, Stillwater, OK 74078-3035 USA

**Keywords:** Interior spruce, Genomic selection, Genotyping-by-sequencing, Open-pollinated families, Genotype x environment interaction, Imputation methods, Multi-trait GS

## Abstract

**Background:**

Genomic selection (GS) in forestry can substantially reduce the length of breeding cycle and increase gain per unit time through early selection and greater selection intensity, particularly for traits of low heritability and late expression. Affordable next-generation sequencing technologies made it possible to genotype large numbers of trees at a reasonable cost.

**Results:**

Genotyping-by-sequencing was used to genotype 1,126 Interior spruce trees representing 25 open-pollinated families planted over three sites in British Columbia, Canada. Four imputation algorithms were compared (mean value (MI), singular value decomposition (SVD), expectation maximization (EM), and a newly derived, family-based k-nearest neighbor (kNN-Fam)). Trees were phenotyped for several yield and wood attributes. Single- and multi-site GS prediction models were developed using the Ridge Regression Best Linear Unbiased Predictor (RR-BLUP) and the Generalized Ridge Regression (GRR) to test different assumption about trait architecture. Finally, using PCA, multi-trait GS prediction models were developed. The EM and kNN-Fam imputation methods were superior for 30 and 60% missing data, respectively. The RR-BLUP GS prediction model produced better accuracies than the GRR indicating that the genetic architecture for these traits is complex. GS prediction accuracies for multi-site were high and better than those of single-sites while multi-site predictability produced the lowest accuracies reflecting type-b genetic correlations and deemed unreliable. The incorporation of genomic information in quantitative genetics analyses produced more realistic heritability estimates as half-sib pedigree tended to inflate the additive genetic variance and subsequently both heritability and gain estimates. Principle component scores as representatives of multi-trait GS prediction models produced surprising results where negatively correlated traits could be concurrently selected for using PCA2 and PCA3.

**Conclusions:**

The application of GS to open-pollinated family testing, the simplest form of tree improvement evaluation methods, was proven to be effective. Prediction accuracies obtained for all traits greatly support the integration of GS in tree breeding. While the within-site GS prediction accuracies were high, the results clearly indicate that single-site GS models ability to predict other sites are unreliable supporting the utilization of multi-site approach. Principle component scores provided an opportunity for the concurrent selection of traits with different phenotypic optima.

**Electronic supplementary material:**

The online version of this article (doi:10.1186/s12864-015-1597-y) contains supplementary material, which is available to authorized users.

## Background

Tree improvement programs are long-term and resource demanding endeavors requiring repeated cycles of selection, breeding and testing. Most of conventional tree breeding programs face major challenges; including, long breeding cycles, large field experiments planted over vast territory, late expression of economic traits (e.g., wood density), and low to medium heritability of traits [1]. The phenotypic selection approach coupled with long testing phase often result in slow accumulation of genetic gain per unit time and cost [2]. Plant breeders adopted Marker-Assisted-Selection (MAS) to take advantage of the linkage disequilibrium (LD) between genetic markers and Quantitative Trait Loci (QTLs) and realized the method’s potential to increase breeding efficiency [3,4]. Similarly, tree breeders perceived MAS as a means to reduce the time required for phenotypic selection, increasing selection intensity, and improving selection precision particularly for low heritability and late expressing traits as well as its ability to overcome major conventional breeding obstacles such as the long and costly breeding cycle [5,6]. However, MAS faced several challenges; as most associations were limited to only specific genetic background due to the rapidly decaying LD in forest trees, the interaction of QTLs effects with the genetic background, the genotype by environment (GxE) interaction, and the fluctuation of the alleles frequency over generations [7]. The complex nature of quantitative traits [8] rendered MAS ineffective in both animal and crop breeding and few successes mostly involving traits with simple inheritance (e.g., disease resistance) were reported [9,10].

Meuwissen et al. [11] introduced Genomic Selection (GS) as a method that collectively uses the genome-wide marker data in predicting the phenotype by estimating the genomic breeding values for each individual. The major advantage of GS is that it does not require the identification of the QTLs or linked markers with target traits as all marker effects are estimated simultaneously and used to develop the prediction model for estimating Genomic Estimated Breeding Values (GEBV) for each individual. Thus, this method is suitable for selection of traits with complex genetic architecture as it does not rely on the identification of a single causal variant, rather it fits the genetic effects of all markers regardless of their known functional relevance [11,12]. In forest tree breeding context, GS has the ability to predict the phenotype for selecting elite genotypes at early age and developmental stage, thus substantially shortening the breeding cycle and increasing the selection differential, ultimately raising the genetic gain per unit time [13-16]. The time savings involve tree testing (for late expressing traits in particular), which is not needed in the next few generations with GS being implemented in the conifer breeding program, thus providing 15–25 years anticipated savings [16].

The development of Next-Generation-Sequencing (NGS) technologies and the implementation of genetic markers from sequence data in quantitative genetics related to GS, the Genomic Best Linear Unbiased Predictor (GBLUP) [17], and the unified single-step evaluation approach (also known as HBLUP, single-step combining pedigree and realized kinship information) [18] have created novel opportunities for breeding, including forest trees [2,19-21]. Genotyping-By-Sequencing (GBS) [22], of the NGS technologies, offers a promising opportunity in studying non-model species including those with large and complex genomes with no assembled reference sequence such as conifers [23]. GBS uses restriction enzymes to allow the sequencing of a reduced subset of the studied genome and the resulting fragments are DNA barcoded to permit multiplexed sequencing. GBS has made genome-wide population studies possible due to the affordability of the method and its capability of resolving tens of thousands of markers scattered throughout the genome.

In this study, using GBS as a genotyping platform, we developed GS prediction models in a dataset of 1,126 Interior spruce trees representing 25 open-pollinated families replicated over three sites in British Columbia (BC), Canada. White and Interior spruce are one of the most economically important forest tree species in BC. Interior spruce is a complex of white spruce (*Picea glauca* (Moench) Voss), Engelmann spruce (*Picea engelmannii* Parry), and their hybrids and, because of their similar growing habitats and silvicultural requirements, they are often collectively treated as one complex [24]. While white spruce shows transcontinental distribution, the natural distribution of Engelmann spruce is much more limited and scattered and in BC province is confined to the northern part of central BC. Hybridization occurs mainly at mid elevations, where their distributions overlap. Recently, extensive genetic and genomic resources became available for this species (4.9 million scaffolds from the 20.8 giga base pairs draft genome of Interior spruce individual PG29, Birol et al. [25]; 21,840 spruce ESTs microarray employed in genetical genomics of interior spruce progenies [26]). The objectives of the present study were to: 1) evaluate the efficiency of GBS as a rapid genetic marker genotyping platform for GS studies, 2) investigate different imputation algorithms for GBS data on GS prediction accuracy, 3) compare two GS approaches (Ridge regression best linear unbiased predictor (RR-BLUP) and generalized ridge regression (GRR)), 4) investigate the heterogeneous GxE effect on GS prediction accuracy in space, and 5) use PCA in the comparisons of multi- vs. single-trait GS prediction models.

## Results

### Genotyping, missing data imputation, and selection of imputation method

In this study, 1,126 38-year-old Interior spruce trees (*Picea glauca* (Moench) Voss x *Picea engelmannii* Parry ex Engelm.) originating from 25 open-pollinated families selected for their superior growth traits were sampled from the progeny test trial planted on three sites, (1) Aleza Lake, (2) Prince George Tree Improvement Station (PGTIS), and (3) Quesnel. A cost-effective NGS technology, genotyping-by-sequencing (GBS), was employed for genotyping a 20GB unassembled genome such as spruce. After two 48-multiplexed sequencing passes, a total of 4,798,791,310 good barcoded reads was generated, and the median of read depth per site was at 3.92 (averaged 4.58 ± 4.28). TASSEL UNEAK SNP calling pipeline was used to determine SNP polymorphism for these 1,126 spruce trees, resulting in a large genotype table of 1,232,406 SNPs [23,27]. Typical to GBS, a low coverage sequence platform, many markers tended to have missing data even after the repeated sequencing of all studied trees (see Discussion, for more details). From the identified 1,232,406 SNPs, the applied imputation methods and filtering (minimum minor allele frequency of 0.05) used produced genotyping files ranged from 8,868 (MI-30% and EM-30%) to 62,618 (kNN-Fam-60%) SNPs (Table 1). Imputation accuracy ranges from 0.77 (SVD 10 iterations) to 0.82 (SVD with 2 iterations). On average, SVD with 2 iterations produced the best accuracy in the four currently existing methods: MI, SVD, EM and kNN. Using K’s (in K-nearest neighbors) from family versus non-family members, accuracy for kNN-Fam imputation ranged from 0.77 to 0.85. In general, including more family members resulted in higher accuracy (Additional file 1); however, imputation accuracy remained unchanged (and did not improve), when the number of non-family members that was included was larger than the family size. The best imputation accuracy gained was at K1 = 5 and K2 = 20, which represented the K values used in this study for imputing the whole SNP table (Additional file 1). As a result, we chose kNN-Fam over kNN of Troyanskaya et al. [28] due to its slight superiority in accuracy. The SNP table imputed with this method is referred to as kNN-Fam.

**Table 1:** **Imputation methods used for genotyping-by-sequencing data**

**Imputation method** ^**1**^	**Missing data threshold**	**Imputation algorithm**	**# of SNPs**
**MI**	30%	Mean imputation (MI)	8,868
**MI**	60%	Mean imputation (MI)	47,521
**EM**	30%	Expectation-maximization (EM)	8,868
**kNN-Fam**	60%	Family-based K-nearest neighbor (kNN-Fam)	62,198
**SVD**	60%	Singular Value Decomposition (SVD)	55,618

^1^See main text for abbreviations.

The selection of specific imputation methods for genomic selection analyses were restricted to the method with greater GS accuracies within the same percentage of missing data class (i.e., 30% vs. 60%). For the 30% missing data, the EM-30% produced greater accuracy than MI-30%, similarly for the 60% missing data, the kNN-Fam-60% and SVD-60% produced better accuracies comparing to MI-60%; however, the kNN-Fam-60% was superior to SVD-60% (see below). This comparison was done based on GS prediction accuracies produced for the two GS models and the seven studied traits for both single- and multi-site scenarios (see below).

### Trait heritabilities

Using genotypes resulting from the EM-30% algorithm imputed data, the narrow-sense heritabilities of the traits estimated from the pedigree (ABLUP, i.e. the conventional BLUP model using the pedigree-based relationship matrix) and genomic best linear unbiased predictors (GBLUP using the genomic-based realized kinship matrix) produced several broad generalizations that include: 1) single- and multi-site heritabilities were higher for ABLUP than those from their GBLUP counterparts, 2) multi-site heritabilities were lower than that of a single site for both ABLUP and GBLUP, 3) trait heritabilities varied among sites for both ABLUP and GBLUP; however, the differences were lower for the GBLUP than that of the ABLUP, 4) the Quesnel site produced higher heritabilities than PGTIS and Aleza Lake, yet they have some overlapping ranges, and 5) standard error estimates of heritabilities obtained from ABLUP were higher than those from GBLUP for single- and multi-site (Table 2). Lower GBLUP heritabilities were expected as ABLUP tended to inflate the estimates as the pedigree based analysis assumptions are often violated due to mating pattern, relatedness built-up due to population history, and inability to separate common environment effect from genetics.

**Table 2:** **Multi- and single site heritability estimates and their standard errors using pedigree (ABLUP) and genomic (GBLUP) best linear unbiased predictors**

**Trait**	**ABLUP**				**GBLUP (EM-30%)**			
	**Multi-site**	**Single site**			**Multi-site**	**Single site**		
		**PGTIS**	**Aleza L.**	**Quesnel**		**PGTIS**	**Aleza L.**	**Quesnel**
**HT**	0.35 ± 0.14	0.64 ± 0.22	0.43 ± 0.19	0.98 ± 0.02	0.20 ± 0.06	0.50 ± 0.15	0.32 ± 0.14	0.56 ± 0.13
**DBH**	0.05 ± 0.08	0.39 ± 0.17	0.28 ± 0.15	0.55 ± 0.19	0.07 ± 0.06	0.37 ± 0.15	0.26 ± 0.13	0.53 ± 0.15
**VOL**	0.09 ± 0.10	0.45 ± 0.18	0.29 ± 0.15	0.76 ± 0.23	0.09 ± 0.06	0.42 ± 0.15	0.27 ± 0.13	0.60 ± 0.15
**V**_**Dir**_	0.28 ± 0.12	0.31 ± 0.15	0.38 ± 0.17	0.78 ± 0.24	0.12 ± 0.06	0.17 ± 0.11	0.37 ± 0.15	0.49 ± 0.14
**WD**_**res**_	0.27 ± 0.12	0.59 ± 0.21	0.65 ± 0.22	0.42 ± 0.15	0.10 ± 0.06	0.49 ± 0.15	0.28 ± 0.13	0.42 ± 0.14
**WD**_**X-ray**_	0.38 ± 0.14	0.55 ± 0.20	0.48 ± 0.19	0.59 ± 0.20	0.18 ± 0.06	0.28 ± 0.13	0.39 ± 0.15	0.43 ± 0.13
**MoE**_**d**_	0.28 ± 0.12	0.31 ± 0.15	0.38 ± 0.17	0.78 ± 0.24	0.12 ± 0.06	0.17 ± 0.11	0.37 ± 0.15	0.49 ± 0.14

Traits are HT: height in m; DBH: diameter at breast height in cm; VOL: stem volume in m3; VDir: acoustic velocity in km/s; WDRes: resistance to drilling; WDX-ray: wood density in kg/m3 using X-ray densitometry; MoEd: dynamic modulus of elasticity.

### Prediction accuracy for different GS models and imputation methods

The accuracy of GS models (RR-BLUP and GRR) in predicting the GEBV were evaluated for the seven studied traits using all imputation methods (30% missing data: MI and EM, and 60% missing data: MI, kNN-Fam, and SVD) and over the four cross-validation scenarios: 1) within each individual site, 2) cross-site (all possible combinations), 3) within multi-site (the three sites combined), and 4) the multi-site population in predicting individual site (see below).

### Within site GS accuracies

**Figure 1:**
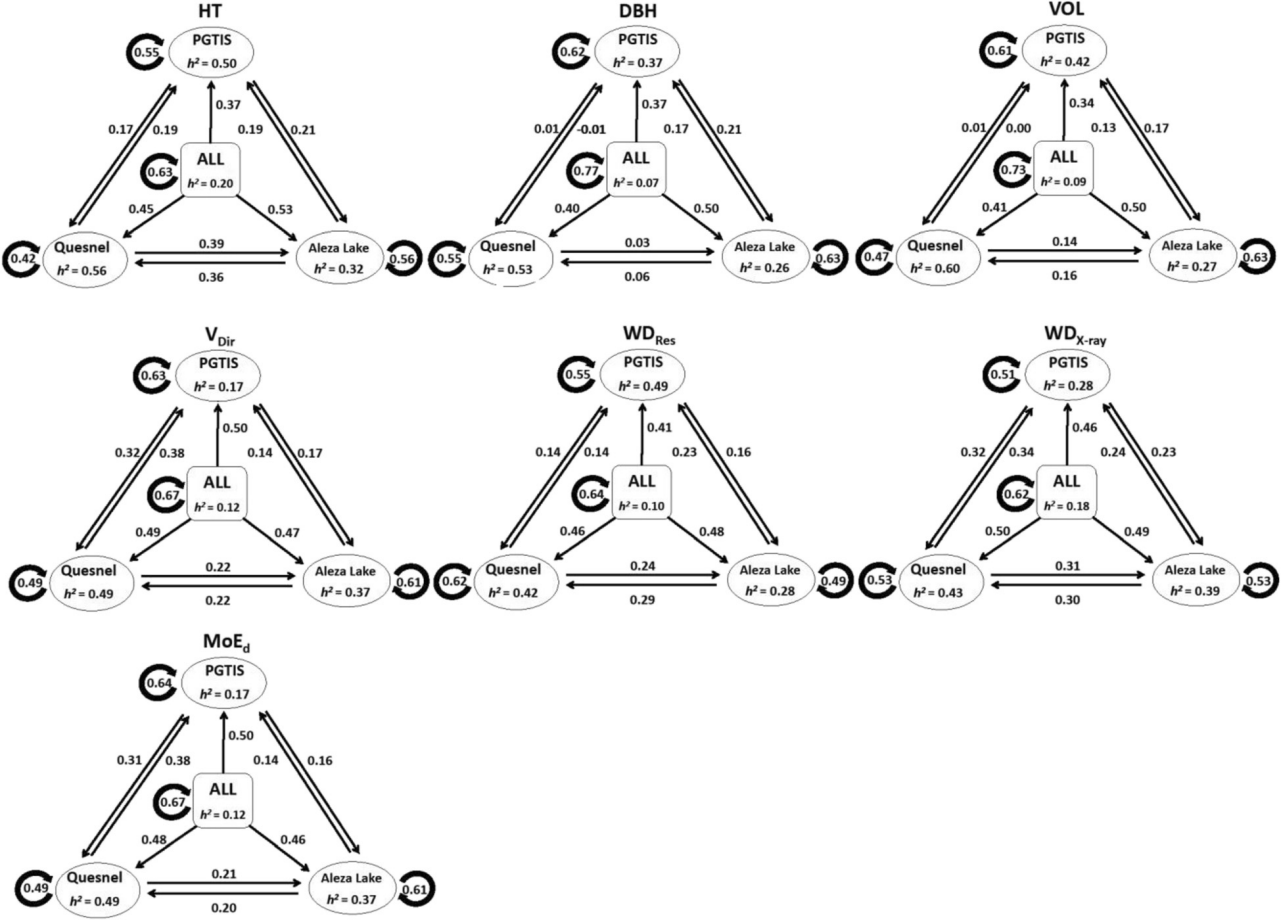
Genomic selection prediction accuracies for each of the seven studied traits using the RR-BLUP model (within single site (three), cross-sites (six), within multi-site (one), and for multi-site to single site (three)), along with narrow-sense heritabilities (*h* ^*2*^) from single- and multi-site GBLUP analyses. Sites are Prince George Tree Improvement Station (PGTIS), Quesnel, Aleza lake, and multi-site (ALL). Traits are HT: height in m; DBH: diameter at breast height in cm; VOL: stem volume in m^3^; V_Dir_: acoustic velocity in km/s; WD_Res_: resistance to drilling; WD_X-ray_: wood density in kg/m^3^ using X-ray densitometry; MoE_d_: dynamic modulus of elasticity.

Across all imputation methods (30% and 60% missing data), the RR-BLUP produced higher within site GEBV accuracies than the GRR (Tables 3 and 4, Figure 1, Additional file 2). In general, the RR-BLUP produced higher accuracies than the GRR (100 out of the possible 105 comparisons for both GS models) and this was also mirrored by their standard error estimates (Tables 3 and 4). Within the 30% missing data imputation methods, the EM-30% produced greater accuracy than MI-30% for all traits for RR-BLUP (traits averages were 0.51, 0.50, and 0.46 as opposed to 0.52, 0.51, and 0.46 for PGTIS, Aleza Lake, and Quesnel sites, respectively) and GRR (averages were 0.49, 0.43, and 0.41 vs. 0.49, 0.46, and 0.41 for PGTIS, Aleza Lake, and Quesnel sites, respectively) (Table 3). The 60% missing data imputation methods produced similar GS prediction and confirmed the superiority of the RR-BLUP over GRR and additionally highlighting the better accuracies for kNN-Fam-60% compared to MI-60% and SVD-60% (Table 4).

**Table 3:** **Within site (PGTIS, Aleza Lake (AL), and Quesnel) genomic selection prediction accuracies and their standard errors for RR-BLUP and GRR models across 30% missing data imputation methods (MI-30% and EM-30%)**

**Trait**	**GS model**	**Imputation method**					
		**MI-30%**			**ME-30%**		
		**PGTIS**	**AL**	**Quesnel**	**PGTIS**	**AL**	**Quesnel**
**HT**	**RR**-**BLUP**	0.48 ± 0.003^1^	0.46 ± 0.002	0.33 ± 0.003	0.50 ± 0.003	0.48 ± 0.003	0.35 ± 0.004
	**GRR**	0.44 ± 0.003	0.45 ± 0.010	0.27 ± 0.007	0.46 ± 0.005	0.45 ± 0.005	0.29 ± 0.006
**DBH**	**RR**-**BLUP**	0.58 ± 0.002	0.55 ± 0.003	0.53 ± 0.004	0.58 ± 0.003	0.55 ± 0.002	0.53 ± 0.003
	**GRR**	0.54 ± 0.003	0.47 ± 0.017	0.51 ± 0.006	0.53 ± 0.004	0.49 ± 0.006	0.51 ± 0.003
**VOL**	**RR**-**BLUP**	0.56 ± 0.002	0.54 ± 0.003	0.44 ± 0.003	0.55 ± 0.004	0.54 ± 0.002	0.45 ± 0.002
	**GRR**	0.52 ± 0.003	0.50 ± 0.004	0.42 ± 0.006	0.53 ± 0.004	0.49 ± 0.004	0.41 ± 0.006
**V**_**Dir**_	**RR**-**BLUP**	0.55 ± 0.002	0.54 ± 0.002	0.41 ± 0.004	0.55 ± 0.003	0.55 ± 0.002	0.41 ± 0.004
	**GRR**	0.52 ± 0.003	0.48 ± 0.004	0.31 ± 0.006	0.52 ± 0.013	0.50 ± 0.005	0.33 ± 0.004
**WD**_**Res**_	**RR**-**BLUP**	0.47 ± 0.003	0.37 ± 0.003	0.59 ± 0.003	0.49 ± 0.003	0.39 ± 0.004	0.59 ± 0.003
	**GRR**	0.46 ± 0.005	0.34 ± 0.005	0.54 ± 0.005	0.44 ± 0.009	0.33 ± 0.007	0.54 ± 0.005
**WD**_**X-ray**_	**RR**-**BLUP**	0.41 ± 0.003	0.49 ± 0.003	0.50 ± 0.002	0.43 ± 0.003	0.48 ± 0.003	0.50 ± 0.001
	**GRR**	0.41 ± 0.004	0.25 ± 0.011	0.50 ± 0.004	0.42 ± 0.003	0.46 ± 0.020	0.50 ± 0.002
**MoE**_**d**_	**RR**-**BLUP**	0.55 ± 0.003	0.55 ± 0.002	0.40 ± 0.004	0.55 ± 0.002	0.55 ± 0.002	0.39 ± 0.003
	**GRR**	0.53 ± 0.004	0.51 ± 0.003	0.30 ± 0.006	0.55 ± 0.004	0.52 ± 0.005	0.29 ± 0.006
**Ave.**	**RR**-**BLUP**	0.51 ± 0.062	0.50 ± 0.067	0.46 ± 0.088	0.52 ± 0.051	0.51 ± 0.060	0.46 ± 0.085
	**GRR**	0.49 ± 0.051	0.43 ± 0.097	0.41 ± 0.113	0.49 ± 0.052	0.46 ± 0.063	0.41 ± 0.108

Traits are HT: height in m; DBH: diameter at breast height in cm; VOL: stem volume in m^3^; V_Dir_: acoustic velocity in km/s; WD_Res_: resistance to drilling; WD_X-ray_: wood density in kg/m^3^ using X-ray densitometry; MoE_d_: dynamic modulus of elasticity.

**Table 4:** **Within site (PGTIS, Aleza Lake (AL), and Quesnel) genomic selection prediction accuracies and their standard errors for RR-BLUP and GRR models across 60% missing data imputation methods (MI-60%, kNN-Fam-60% and SVD-60%)**

**Trait**	**GS model**	**Imputation method**								
		**MI-60%**			**kNN-Fam-60%**			**SVD-60%**		
		**PGTIS**	**AL**	**Quesnel**	**PGTIS**	**AL**	**Quesnel**	**PGTIS**	**AL**	**Quesnel**
**HT**	**RR-BLUP**	0.54 ± 0.002	0.51 ± 0.003	0.40 ± 0.002	0.55 ± 0.002	0.56 ± 0.002	0.42 ± 0.002	0.53 ± 0.003	0.50 ± 0.004	0.42 ± 0.003
	**GRR**	0.51 ± 0.005	0.45 ± 0.011	0.34 ± 0.007	0.51 ± 0.005	0.51 ± 0.006	0.39 ± 0.005	0.51 ± 0.004	0.47 ± 0.006	0.37 ± 0.005
**DBH**	**RR-BLUP**	0.62 ± 0.002	0.60 ± 0.002	0.56 ± 0.003	0.62 ± 0.001	0.63 ± 0.002	0.55 ± 0.002	0.60 ± 0.002	0.59 ± 0.003	0.54 ± 0.003
	**GRR**	0.59 ± 0.009	0.58 ± 0.004	0.53 ± 0.006	0.59 ± 0.005	0.62 ± 0.004	0.53 ± 0.004	0.59 ± 0.002	0.57 ± 0.004	0.52 ± 0.004
**VOL**	**RR-BLUP**	0.60 ± 0.002	0.58 ± 0.003	0.49 ± 0.003	0.61 ± 0.002	0.63 ± 0.001	0.47 ± 0.002	0.59 ± 0.002	0.57 ± 0.003	0.48 ± 0.003
	**GRR**	0.58 ± 0.005	0.55 ± 0.006	0.44 ± 0.009	0.58 ± 0.003	0.59 ± 0.005	0.44 ± 0.005	0.58 ± 0.003	0.56 ± 0.004	0.45 ± 0.005
**V**_**Dir**_	**RR-BLUP**	0.62 ± 0.002	0.57 ± 0.002	0.46 ± 0.003	0.63 ± 0.002	0.61 ± 0.002	0.49 ± 0.002	0.58 ± 0.002	0.55 ± 0.002	0.46 ± 0.003
	**GRR**	0.59 ± 0.005	0.51 ± 0.010	0.40 ± 0.006	0.60 ± 0.003	0.57 ± 0.005	0.46 ± 0.006	0.57 ± 0.004	0.53 ± 0.003	0.42 ± 0.004
**WD**_**Res**_	**RR-BLUP**	0.53 ± 0.002	0.44 ± 0.002	0.62 ± 0.002	0.55 ± 0.002	0.49 ± 0.002	0.62 ± 0.002	0.56 ± 0.003	0.46 ± 0.004	0.58 ± 0.002
	**GRR**	0.46 ± 0.007	0.36 ± 0.009	0.58 ± 0.004	0.47 ± 0.005	0.44 ± 0.007	0.59 ± 0.005	0.54 ± 0.003	0.43 ± 0.005	0.56 ± 0.003
**WD**_**X-ray**_	**RR-BLUP**	0.49 ± 0.002	0.51 ± 0.002	0.53 ± 0.003	0.51 ± 0.002	0.53 ± 0.002	0.53 ± 0.002	0.50 ± 0.002	0.50 ± 0.002	0.50 ± 0.003
	**GRR**	0.45 ± 0.006	0.47 ± 0.005	0.49 ± 0.009	0.48 ± 0.005	0.50 ± 0.006	0.48 ± 0.009	0.49 ± 0.005	0.49 ± 0.003	0.49 ± 0.004
**MoE**_**d**_	**RR-BLUP**	0.62 ± 0.001	0.57 ± 0.002	0.45 ± 0.002	0.64 ± 0.001	0.61 ± 0.001	0.49 ± 0.002	0.59 ± 0.003	0.54 ± 0.004	0.45 ± 0.004
	**GRR**	0.60 ± 0.003	0.52 ± 0.007	0.38 ± 0.007	0.61 ± 0.004	0.58 ± 0.004	0.45 ± 0.004	0.58 ± 0.002	0.52 ± 0.004	0.41 ± 0.005
**Ave.**	**RR**-**BLUP**	0.57 ± 0.053	0.54 ± 0.056	0.50 ± 0.074	0.59 ± 0.050	0.58 ± 0.054	0.51 ± 0.064	0.56 ± 0.037	0.53 ± 0.045	0.49 ± 0.055
	**GRR**	0.54 ± 0.065	0.49 ± 0.073	0.45 ± 0.086	0.55 ± 0.060	0.54 ± 0.063	0.48 ± 0.065	0.55 ± 0.039	0.51 ± 0.050	0.46 ± 0.067

Traits are HT: height in m; DBH: diameter at breast height in cm; VOL: stem volume in m^3^; V_Dir_: acoustic velocity in km/s; WD_Res_: resistance to drilling; WD_X-ray_: wood density in kg/m^3^ using X-ray densitometry; MoE_d_: dynamic modulus of elasticity.

### Multi-sites GS accuracies

**Figure 2:**
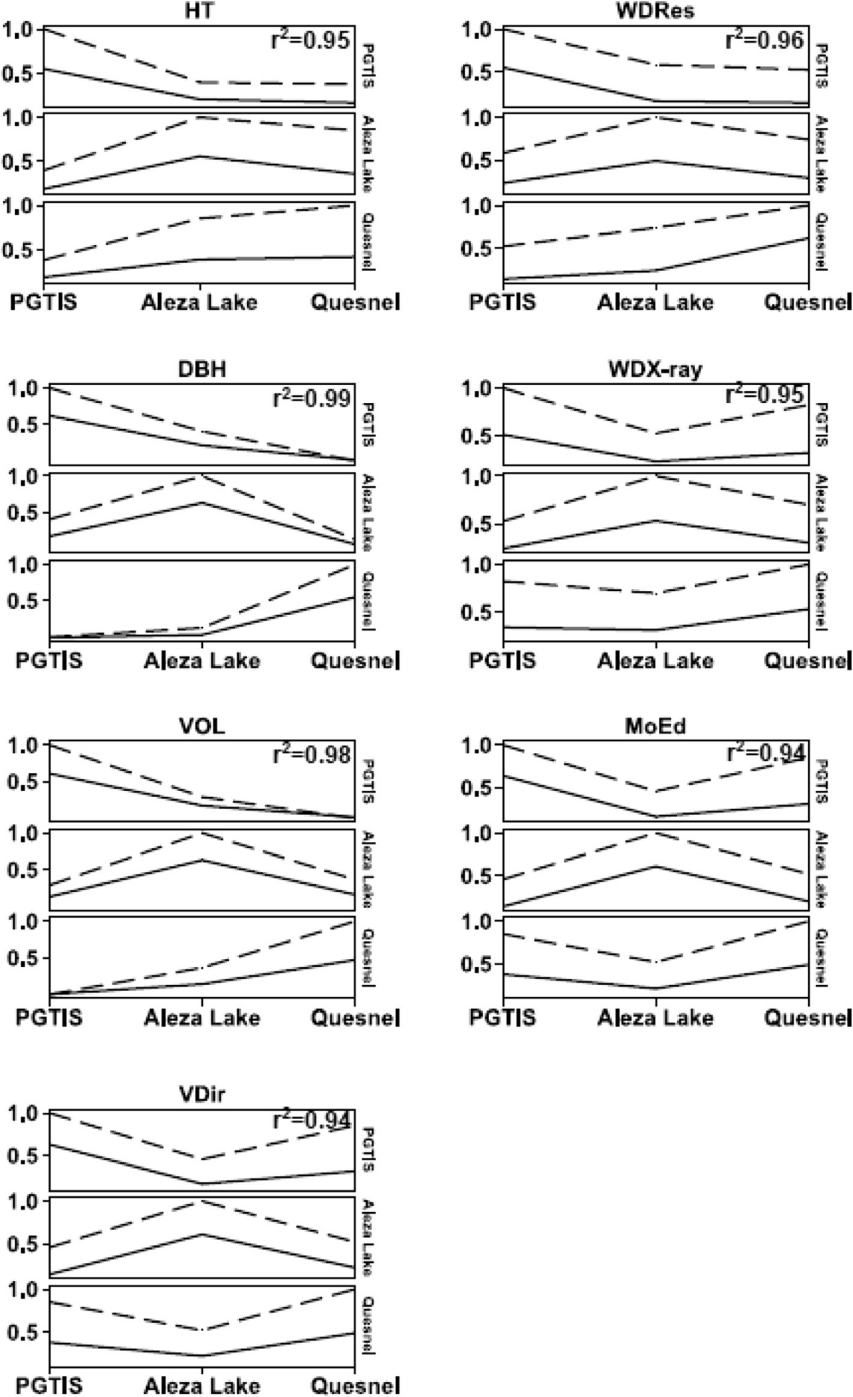
Accuracy of cross-population GS prediction models (indicating their respective correlations (Y-axis)) for seven growth and wood quality traits for interior spruce. Sites are Prince George Tree Improvement Station (PGTIS), Quesnel, and Aleza lake. Traits are HT: height in m; DBH: diameter at breast height in cm; VOL: stem volume in m^3^; V_Dir_: acoustic velocity in km/s; WD_Res_: resistance to drilling; WD_X-ray_: wood density in kg/m^3^ using X-ray densitometry; MoE_d_: dynamic modulus of elasticity. Dash and solid lines represent Type B correlation and prediction accuracy, respectively.

Unlike within site cross-validation, testing the applicability of a GS model for a specific site to predict the GEBV of other sites generally produced lower accuracies for both models (RR-BLUP and GRR) (Figure 1, Additional files 2 and 3). This is expected due to the GxE interaction even when the three sites are located within one breeding zone (Prince George Seed Planning Zone (http://www.for.gov.bc.ca/hfd/pubs/docs/mr/annual/ar_1995-96/pspzm.htm)). For simplicity, in this section we will restrict the cross-sites comparisons to the imputation method with the highest number of SNPs (i.e., kNN-Fam-60% (62,198 SNPs)), and the GS model with highest accuracies (i.e., RR-BLUP (Additional file 2)). Over the seven studied traits, the RR-BLUP model produced cross-site validation accuracies ranging from 0.16 and 0.23 when PGTIS was used to predict the GEBV of Aleza Lake (1→2), 0.13 and 0.24 for 2→1, 0.01 and 0.32 for PGTIS to predict Quesnel (1→3), 0.0 and 0.38 for 3→1, 0.06 and 0.36 for 2→3, and 0.03 and 0.39 for 3→2 (Additional files 2 and 3). The estimated type-b genetic correlations between sites mimicked the trend observed for cross sites GS accuracy with their Pearson-product-moment correlations ranging between 0.94 and 0.99 (*P* < 0.05) over the seven studied traits for the kNN-Fam-60% imputation method (Figure 2).

### Within multi-site GS accuracies

Similar to within site assessment, the within multi-site cross-validation produced higher GEBV accuracies for RR-BLUP as compared to GRR and this increase in accuracy persisted across all 30% and 60% missing data imputation methods (Table 5). Comparisons between imputation methods revealed that EM-30% and kNN-Fam-60% produced better accuracies (Table 5, Figure 1, Additional file 2). Again, we will restrict the GEBV accuracy comparisons to the kNN-Fam-60% imputation method as it uses the largest number of SNPs (62,198 SNPs). On average and across the seven studied traits, GS accuracies ranged between 0.62 and 0.77 for both RR-BLUB and GRR (Table 5). The span of this range is far greater than the one observed within sites and cross-sites validation (Tables 2, 3 and 4). These estimates represent the most realistic accuracies as they accommodated the GxE interaction and, furthermore, were produced with a large training population size (90% of the total N = 1,126).

**Table 5:** **Multi-site genomic selection prediction accuracies and their standard errors for RR-BLUP and GRR models for the studied five imputation methods**

**Trait**	**GS model**	**Imputation method**				
		**MI**-**30%**	**EM**-**30%**	**MI**-**60%**	**kNN-Fam**-**60%**	**SVD**-**60%**
**HT**	**RR-BLUP**	0.56 ± 0.001^3^	0.58 ± 0.001	0.60 ± 0.001	0.63 ± 0.001	0.61 ± 0.001
	**GRR**	0.50 ± 0.002	0.48 ± 0.004	0.57 ± 0.003	0.62 ± 0.002	0.58 ± 0.002
**DBH**	**RR-BLUP**	0.71 ± 0.001	0.72 ± 0.001	0.75 ± 0.001	0.77 ± 0.001	0.76 ± 0.001
	**GRR**	0.71 ± 0.001	0.73 ± 0.001	0.74 ± 0.001	0.77 ± 0.001	0.75 ± 0.001
**VOL**	**RR-BLUP**	0.67 ± 0.001	0.68 ± 0.001	0.71 ± 0.001	0.73 ± 0.001	0.72 ± 0.001
	**GRR**	0.67 ± 0.001	0.68 ± 0.001	0.70 ± 0.001	0.72 ± 0.001	0.71 ± 0.001
**V**_**Dir**_	**RR-BLUP**	0.59 ± 0.001	0.61 ± 0.001	0.63 ± 0.001	0.67 ± 0.001	0.65 ± 0.001
	**GRR**	0.52 ± 0.004	0.50 ± 0.003	0.62 ± 0.002	0.66 ± 0.001	0.62 ± 0.006
**WD**_**Res**_	**RR-BLUP**	0.56 ± 0.001	0.58 ± 0.001	0.62 ± 0.001	0.64 ± 0.001	0.63 ± 0.001
	**GRR**	0.48 ± 0.002	0.47 ± 0.003	0.59 ± 0.003	0.64 ± 0.002	0.60 ± 0.003
**WD**_**X-ray**_	**RR-BLUP**	0.55 ± 0.001	0.56 ± 0.001	0.59 ± 0.001	0.62 ± 0.001	0.61 ± 0.001
	**GRR**	0.54 ± 0.002	0.55 ± 0.001	0.59 ± 0.002	0.62 ± 0.001	0.60 ± 0.002
**MoE**_**d**_	**RR-BLUP**	0.50 ± 0.001	0.61 ± 0.001	0.63 ± 0.001	0.67 ± 0.001	0.65 ± 0.001
	**GRR**	0.50 ± 0.013	0.56 ± 0.002	0.63 ± 0.002	0.66 ± 0.001	0.64 ± 0.002
**Ave.**	**RR-BLUP**	0.59 ± 0.073	0.62 ± 0.059	0.65 ± 0.060	0.68 ± 0.055	0.66 ± 0.057
	**GRR**	0.56 ± 0.091	0.57 ± 0.101	0.63 ± 0.063	0.67 ± 0.056	0.64 ± 0.063

Traits are HT: height in m; DBH: diameter at breast height in cm; VOL: stem volume in m^3^; V_Dir_: acoustic velocity in km/s; WD_Res_: resistance to drilling; WD_X-ray_: wood density in kg/m^3^ using X-ray densitometry; MoE_d_: dynamic modulus of elasticity.

### Single- vs. multi-site accuracies

When the meta-population was used to predict the GEBV for each individual site, the observed accuracies were high with Aleza Lake producing the highest accuracies (average over the 7 traits of 0.49 for RR-BLUP and GRR) followed by Quesnel (averages of 0.46 and 0.45 for RR-BLUP and GRR, respectively) and PGTIS which produced the lowest accuracies (average of 0.42 for both RR-BLUP and GRR) (Table 6). These accuracies are higher than those observed for the cross-site validation (Table 6, Figure 1, Additional file 2).

**Table 6:** **Single site GS prediction accuracies and their standard errors resulting from using the multi-sites as training population for RR**-**BLUP and GRR models for kNN**-**Fam**-**60% imputation method**

**Traits**	**GS model**	**Cross-validation**			
		**Multi-sites**	**PGTIS**	**Aleza Lake**	**Quesnel**
**HT**	**RR**-**BLUP**	0.63 ± 0.001	0.37 ± 0.001	0.53 ± 0.002	0.45 ± 0.001
	**GRR**	0.62 ± 0.002	0.36 ± 0.003	0.52 ± 0.003	0.45 ± 0.002
**DBH**	**RR**-**BLUP**	0.77 ± 0.001	0.37 ± 0.001	0.50 ± 0.001	0.40 ± 0.001
	**GRR**	0.77 ± 0.001	0.37 ± 0.002	0.50 ± 0.001	0.40 ± 0.001
**VOL**	**RR**-**BLUP**	0.73 ± 0.001	0.34 ± 0.001	0.50 ± 0.001	0.41 ± 0.001
	**GRR**	0.72 ± 0.001	0.34 ± 0.002	0.50 ± 0.002	0.40 ± 0.002
**V**_**Dir**_	**RR**-**BLUP**	0.67 ± 0.001	0.50 ± 0.001	0.47 ± 0.001	0.49 ± 0.001
	**GRR**	0.66 ± 0.001	0.49 ± 0.001	0.47 ± 0.001	0.48 ± 0.002
**WD**_**Res**_	**RR**-**BLUP**	0.64 ± 0.001	0.41 ± 0.001	0.48 ± 0.001	0.46 ± 0.001
	**GRR**	0.64 ± 0.002	0.41 ± 0.002	0.48 ± 0.002	0.45 ± 0.003
**WD**_**X-ray**_	**RR**-**BLUP**	0.62 ± 0.001	0.46 ± 0.001	0.49 ± 0.002	0.50 ± 0.001
	**GRR**	0.62 ± 0.001	0.46 ± 0.002	0.49 ± 0.002	0.50 ± 0.002
**MoE**_**d**_	**RR**-**BLUP**	0.67 ± 0.001	0.50 ± 0.001	0.46 ± 0.001	0.48 ± 0.001
	**GRR**	0.66 ± 0.001	0.49 ± 0.002	0.45 ± 0.002	0.47 ± 0.002
**Ave.**	**RR**-**BLUP**	0.68 ± 0.055	0.42 ± 0.066	0.49 ± 0.023	0.46 ± 0.039
	**GRR**	0.67 ± 0.056	0.42 ± 0.063	0.49 ± 0.023	0.45 ± 0.038

Traits are HT: height in m; DBH: diameter at breast height in cm; VOL: stem volume in m^3^; V_Dir_: acoustic velocity in km/s; WD_Res_: resistance to drilling; WD_X-ray_: wood density in kg/m^3^ using X-ray densitometry; MoE_d_: dynamic modulus of elasticity.

### Multi-trait GS prediction models

The first three principle components, PCA1-3, collectively accounted for 86% of the total phenotypic variation and individually accounted for 44, 25, and 17%, respectively. PCA1 produced significant (*P* < 0.002 - 0.0001) loading for all the studied traits and was positive for height (HT) (0.69), diameter at breast height (DBH) (0.80), and acoustic velocity (V_Dir_) (0.09) and negative for wood density using X-ray densitometry (WD_X-ray_) (−0.71) and wood density using resistance to drilling (WD_Res_) (−0.75). PCA2 produced interesting results with significant (*P* < 0.0001) and positive loadings for HT (0.39), V_Dir_ (0.92), and WD_X-ray_ (0.49). Similarly, PCA3 produced significant (*P* < 0.0001) and positive loadings for HT (0.46), DBH (0.38), WD_X-ray_ (0.19) and WD_Res_ (0.64). The fact that growth and wood quality traits produced significant and positive loadings, even if it is for PCA2 and PCA3, is interesting as it creates concurrent selection opportunities for yield and wood quality traits that are commonly known to be negatively correlated. The two GS models produced high prediction accuracies for PCA1 with 0.72 ± 0.001 and 0.71 ± 0.001 for RR-BLUP and GRR, respectively. Similar results were observed for PCA 2 (RR-BLUP: 0.65 ± 0.001 and GRR: 0.64 ± 0.001) and PCA3 (RR-BLUP: 0.57 ± 0.001 and GRR: 0.55 ± 0.002) using the multi-site GS model.

### ABLUP vs. GBLUP elite genotype selection comparison

**Figure 3:**
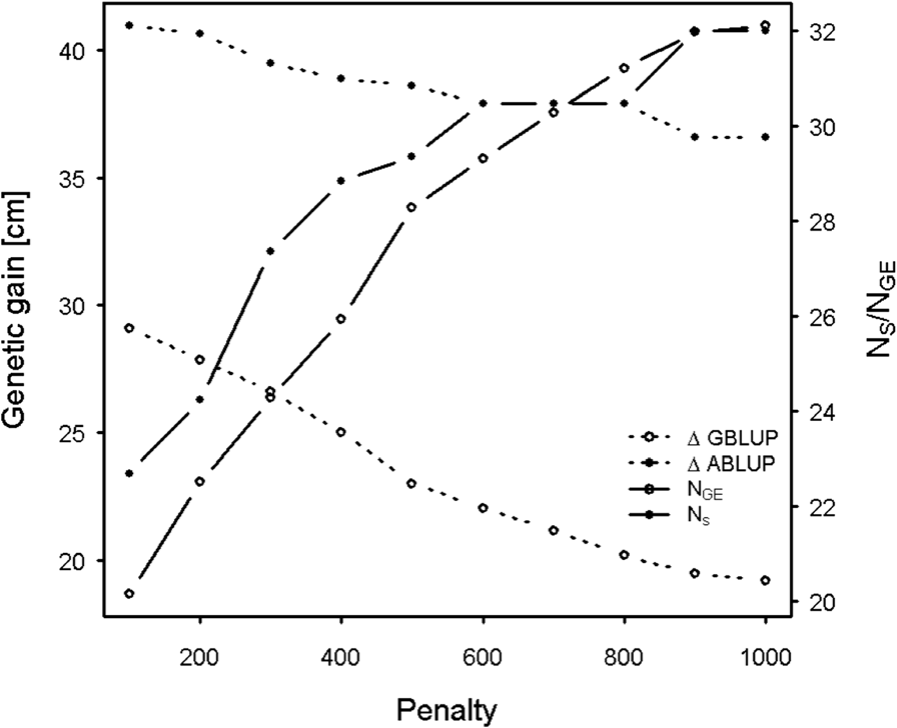
The relationship between height genetic gain and genetic diversity for ABLUP (status number (N_s_)) and GBLUP (number of founder genome equivalent (N_GE_)) across a range of co-ancestry penalties.

Expectedly, across all the range of genetic gain penalties, the selection of 40 elite individuals yielded ABLUP genetic gain higher than that of the GBLUP with percentage increase between 9.2 and 14.6% for 100 and 1,000 penalty classes, respectively (Figure 3). Naturally, any increase in co-ancestry is associated with increase in genetic gain; however, the GBLUP offers greater flexibility for elite genotype selection than the ABLUP as the effective number of genomic equivalent provides a continuum for selection as opposed to the pedigree-based status number which offers only two options of relatedness (unrelated or half-sibs).

## Discussion

### GBS and imputation methods

The utilization of NGS technology, and GBS in particular, provides a low cost opportunity for genomic studies for non-model species [23]. In the present study, GBS produced exceedingly large number of SNPs (1,232,406); however, the low coverage nature of the technique has substantially reduced the available SNPs for analyses due to missing data. Missing data could also result from either the absence of the restriction site in the genomic sequence or due to technical issues associated with DNA digestion or PCR amplification [29,30]. Out of the five imputation methods used, the expectation maximization (EM-30%: [31]) and the newly developed half-sib family-based k-nearest neighbor (kNN-Fam-60%) method resulted in 8,868 and 62,198 SNPs, respectively, and produced the greatest accuracies (Figure 1, for kNN-Fam-60%). We used the EM-30% imputation method in estimating the trait heritabilities employing the GBLUP approach [17], while all described imputation methods were used to evaluate the GS models across all described scenarios. We believe that the higher GEBV accuracies attained from the kNN-Fam imputation method are attributable to the method’s capacity of recovering resemblance among individuals within families. In addition, kNN-Fam method proportionately weights family structure and the underlying LD of SNPs, which is also likely contributing to the slightly higher predictability due to its strength of simultaneously capturing identical-by-state with the variants in LD with the causal genes [32].

### Heritability estimates

Treating the offspring from open-pollinated families as half-sibs is often associated with inflated heritability estimates, resulting in an exaggeration of the expected genetic gain [33-35]. In the present study, heritability estimates obtained from the ABLUP were higher than those from the GBLUP (Table 2), highlighting the advantages of incorporating genomic information in standard quantitative genetic analyses [17] to obtain realistic estimates of breeding values and genetic gain (see ABLUP vs. GBLUP elite genotype selection comparison below).

Our results are similar to those reported for another open-pollinated white spruce progeny trial in Québec, Canada [16].While heritability estimates were population-specific, slight differences in GBLUP-based heritability estimates for wood density (WD_X-ray_) and height (36- vs. 22-year-old height) were observed between the two studies (wood density: 0.18 vs. 0.24 and height: 0.20 vs. 0.16) [16]. Additionally, our results suggest that the trait heritability has only limited effect on the prediction accuracy (PA) as diameter at breast height (DBH) and stem volume (VOL) showed high multi-site RR-BLUP predictability despite their low heritability estimates (DBH: h^2^ = 0.07 and PA = 0.77; VOL: h^2^ = 0.09 and PA = 0.73), results consistent with those reported for loblolly pine (*Pinus taeda*) [15,36].

### GS models

GS models suffer from the “large *p*, small *n*” problem, where the number of predictor effects *p* exceeds by far the number of observations *n* (*p*> > *n*). A variety of statistical methods were proposed to handle this issue and they can be classified into three major categories: shrinkage models, Bayesian methods (including variable selection), and semi- or non-parametric methods such as support vector regression and random forest regression. Those methods are different in their assumptions regarding the genetic architecture of the tested traits [1,37]. RR-BLUP, the most common shrinkage model, assumes that the trait is controlled by many genes each with small effects, thus is suitable for traits following the infinitesimal model [8]. RR-BLUP assumes that all marker effects are random, normally, and identically distributed and have a common variance, thus all the effects will be equally shrunken toward zero [1,37,38]. This approach was described previously by Meuwissen et al. [11] and termed SNP-BLUP. In GS and genome wide association studies (GWAS), it is not realistic to use common shrinkage effects for all fitted SNPs across the genome as not all markers will be linked to functional genes and not all gene effects are normally distributed [11]. To overcome this assumption, the Bayesian methods were developed to provide more flexibility in modeling oligogenic traits (i.e., traits that are controlled by few genes each with large effects) [37]; however, these methods are computationally demanding [39]. A new, fast, deterministic, and flexible Ridge regression method was suggested by Shen et al. [38] known as the generalized Ridge regression (GRR). The main difference between RR-BLUP and GRR is that a SNP-specific shrinkage will be used instead of the common shrinkage effect [38], which is more realistic and more suitable to model oligogenic traits and represents a viable alternative to Bayesian models [28].

Our results showed that GRR produced either similar or even lower prediction accuracies as compared to RR-BLUP, which indicates that marker selection by giving different degree of penalization through the application of different shrinkage effects is inadequate for the tested traits. This provides evidence that the tested traits (growth and wood quality) follow the infinitesimal model. Moreover, experimental results in both plants and animals suggested that RR-BLUP provides the best adjustment/compromise between the computational effort and the prediction efficiency [37]. This supports the notion that most of the economically important traits are complex and quantitative in nature (i.e., follow the infinitesimal model). For example, in loblolly pine, Resende et al*.* [14] evaluated RR-BLUP and three Bayesian models across 17 traits related to growth, development, and fusiform rust resistance and the resulting prediction accuracies were marginally different across the four models, except for rust resistance, an oligogenic trait, where the Bayes A and C models resulted in moderately larger performance than RR-BLUP.

### Cross-validation

The multi-site cross-validation produced higher prediction accuracies as compared to single-sites (Tables 3, 4 and 5, Figure 1) as the multi-site training population is three times larger than any of the single-site models, resulting in more accurate estimation of marker effects and this is consequently reflected in higher prediction accuracy and precision [1,37]. Previous GS studies conducted on plant and animal populations clearly demonstrated the role of training population size on prediction accuracy and illustrated the importance of the training population size as compared to the number of markers used in the models, thus supporting the present study results [40-42]. In forestry context, our results are also consistent with prediction accuracies obtained for growth and wood quality attributes in loblolly pine and Eucalyptus [13,15,43]. However, comparing the prediction accuracies between our study and those from the Québec white spruce open-pollinated progeny trial is of interest as the experimental settings were somewhat similar [16]. Height, wood density, and dynamic modulus of elasticity were common traits between the two studies; however, their prediction accuracies were lower than in the present study (height: 0.17 vs. 0.63, wood density: 0.33 vs. 0.64, dynamic modulus of elasticity: 0.21 vs. 0.67). In general, the lower prediction accuracies in the Québec study across all the traits compared to our and other tree species studies, is mainly due to the considerably larger number of tested families (214 vs. 25 families) which resulted in higher *N*_*e*_ (effective population size). It is also worth mentioning that we used the EBV as opposed to the raw phenotype in training our GS models; this could have also contributed to the observed differences.

### Cross-site validation

The economic and ecological importance of interior spruce to British Columbia promoted thorough understanding of the various ecological regions of the species and subsequently 6 unique Seed Planning Zones (SPZs) were identified (Bukley Valley, East Kootenay, Nelson, Prince George, Peace River, and Thompson Okanagan). To date, most forestry GS studies were conducted within the confines of a single “environment model” similar to those GS studies conducted in animal breeding programs where the assumption of a common environment was invoked. The assumption of “common environment” is not suitable in forestry as estimates of GxE, even within a single breeding zone, are high [44] and this motivated breeders to evaluate the performance of a specific genotype or family across different environments to identify generalists for their inclusion in seed production populations [45]. For the successful implementation of GS in tree breeding, it is essential that GS models remain accurate across sites, at least within the dedicated breeding zone. Only two out of the published four GS studies in forest tree tested GxE interaction, these include loblolly pine [14] and white spruce [16]. In the present study, we used data from three sites within the Prince George breeding zone and the observed prediction accuracies of a single site to predict another site were generally low (Additional file 2, Figure 1). The observed reduced prediction accuracies across sites were lower than those obtained from the white spruce and loblolly pine studies. Thus, it is important to pay considerable attention to the structure of the training population; hence the developed models reflect the underpinning forces affecting trait expression and their response to sites heterogeneities.

### Multi-trait GS prediction models

GS models are trait-specific and do not lend themselves to multi-trait selection as does index selection method which maximizes the correlation between the index score of an individual and its breeding value [46]. Yet, selection indices require prior knowledge about the economic value of the traits for proper scaling before optimum phenotypic weights can be estimated. The use of Principle Component Analysis offered an opportunity to handle a set of correlated variables by reducing the dimensionality to a set of uncorrelated ones (i.e., principal components). Negative genetic correlations between yield and wood quality traits are commonly observed [47] and the results from PC1 which accounts for 44% of the total variation confirmed these observations. However, while yield and wood quality are known to act in antagonizing fashion, the results based on PC2 and PC3, albeit collectively accounting for 42% of the total variation, created interesting opportunities for the concurrent selection for both traits without any adverse effect associated with the known negative correlations. It seems that PC2 and PC3 accessed different combinations of SNPs (i.e., causal genes) that work in the same direction. While we did not consider any prior economic knowledge for weighing in constructing the PCs, the results from PC2-3 clearly demonstrated that it is (to a certain extent) also possible to artificially co-select such attributes that are commonly known to be negatively correlated in the same positive direction. Considering economic weights for traits during constructing selection indices can result in changing the magnitude of genetic correlation among these traits as a consequence of selection. This change in genetic correlation is expected to change SNP effects and thus frequent training is required for GS model to be effective over generations. Finally, our objective of using PCA is to offer a simple method that accounts for the inter-relation (genetic correlation) between the studied traits and provide an opportunity for further expansions that consider economic weights.

### ABLUP vs. GBLUP elite genotype selection comparison

The observed genetic gain differences between the ABLUP and GBLUP across all co-ancestry penalties were not surprising as heritability, breeding value of an individual, and genetic gain estimates are expected to be higher in open-pollinated populations due to the ABLUP inability to ascertain the true genetic relationship among offspring [33-35]. On the other hand, GBLUP relies on estimating the realized kinship which provides a more accurate ascertainment of the genealogical relationships among members of an open-pollinated family and thus, resulting in more realistic gain estimates due to adjustment for Mendelian sampling term [48]. Our results are similar to those reported in the Québec white spruce study as they consistently produced higher gains from pedigree- vs. marker-based methods [16].

It should be pointed out that the Bulmer effect (i.e., reduction in response to selection) would be similar for ABLUP and GBLUP and thus the response to selection for both methods will be similarly affected irrespective of the breeding values estimation method used [49]. If genomic selection effectively reduces generation interval, then in the forestry context, a relatively smaller reference (training) population size is needed to attain the same response to selection from larger traditional population (i.e., ABLUP). Conversely, if generation turnover is not possible, then larger training population size is required, therefore defeating GS goals. Bastiaansen et al. [50] found similar response to selection for GBLUP and ABLUP but the former accumulated lower level of inbreeding and consequently higher genetic variance than the latter.

### Genomic selection in forestry

Open-pollinated family testing is a formidable and economically viable option for screening a larger number of candidate parents without the development of “structured pedigree” that represents the backbone of most conventional tree breeding methods. The simplicity of the method made it an attractive first step before starting a full-blown tree improvement program. Indeed, this was the case for the New Zealand radiata pine (*Pinus radiata*) breeding program as open-pollinated testing provided a quick and inexpensive screening method [51] and subsequently the selected parents were included in a full pedigree-based breeding program [52]. However, the commonly used assumption of treating open-pollinated offspring as half-sib family is by far the greatest drawback of this method as most genetic parameters (e.g., breeding values, trait heritabilities, and gain estimates) are upwardly biased and this was clearly demonstrated in many studies including the present one [16]. The introduction of genomic data (e.g., SNP markers) has provided the means to overcome this drawback and the genealogical relationship among open-pollinated family members is clearly and accurately ascertained. At present, many open-pollinated family testing trials have reached an advanced age and are often abandoned, though they could provide badly needed information for late expressed traits that could not be obtained from younger conventional trials. The present study and that of Beaulieu at al. [16] provided examples of producing yield and wood quality attributes data with unprecedented accuracy and this became possible through the integration of genomic information in the quantitative genetic analyses (e.g., ABLUP vs. GBLUP).

In the present study, the accuracy of predicting breeding values varied across the different studied population scales with within multi-site being the highest and cross sites being the lowest (Figure 1). The high within multi-site GS prediction accuracies offer an opportunity to obtain reliable results for difficult traits such as wood density and yield and points towards considering “old” open-pollinated tests as a valuable source for information. The developed prediction models could be used for selecting elite genotypes with unprecedented selection intensity for their inclusion in future seed production populations, and this can be accomplished without the creation of a single cross.

In the present study, GBS successfully provided the information for genomic-based quantitative genetics analyses at reasonable cost. To our knowledge, this study represents the first large-scale use of GBS in a forest tree species known to a have complex genome and for which no reference sequence has been assembled yet (N = 1,126 trees). It is noteworthy to mention that this study was initiated before the release of Norway and white spruce genome sequences [53,25]. However, as the assemblies of the two spruce genomes are not anchored and ordered along the chromosomes, there is little advantage over de novo SNP markers.

## Conclusions

The results reported here suggest that GBS can be used as a genotyping platform for the application of GS in forestry. The utilization of proper imputation algorithms is needed to overcome the commonly observed problem of missing data with GBS. Greater GS prediction accuracies were obtained for RR-BLUP as compared to GRR indicating that the studied traits follow the infinitesimal model. Greater accuracies were obtained for multi-site GS model and points to the inherent lack of reliability for cross-site prediction. The utilization of principle component analysis as a multi-trait GS approach was proven effective in dealing with negatively correlated traits.

## Methods

### Experimental population and DNA sampling

For this study, 1,126 38-year-old Interior spruce trees (*Picea glauca* (Moench) Voss x *Picea engelmanni*i Parry ex Engelm.) were sampled from a progeny test trial established by the Ministry of Forests, Lands and Natural Resource Operations of British Columbia Canada, and planted on three sites [Aleza Lake (Lat. 54° 03′ 15.7″ N, Long. 122° 06′ 35.4″ W, Elev. 700 mas), Prince George Tree Improvement Station (PGTIS) (Lat. 53° 46′ 17.9″ N, Long. 122° 43′ 07.6″W, Elev. 610 mas), and Quesnel (Lat. 52° 59′ 27.2″ N, Long. 122° 12′ 30.6″ W, Elev. 915 mas)]. The sites were established in 1972/73 and consisted of 181 open-pollinated families using 3-year-old seedlings planted at 2.5×2.5 m spacing in a complete randomized block design with five or ten blocks and ten or fifteen tree-row-plots, respectively. Twenty-five families were selected based on their superior growth traits and four trees per family from four blocks per site were randomly sampled (maximum of 32 trees per family). Evidence of similar genetic diversity between selected and unselected populations have been reported for spruces, including white spruce [54,55]. The differences across all the three sites in the relationship between overall X-ray density and growth traits (see below) indicated that the Quesnel site is most favorable while PGTIS least favorable for growing interior spruce (YA El-Kassaby, pers. obs.).

### Genotyping and SNP selection

DNA extraction was performed on dormant vegetative buds of the sampled trees using a CTAB procedure modified after Doyle and Doyle [56]. To generate a high-density SNP profile for the 1,126 spruce DNA extracts, we conducted a multiplexed, high-throughput Genotyping-by-Sequencing (GBS) following Elshire et al. [22] and Chen et al. [23]. A 48-plex GBS library comprising of 47 DNA samples and a negative control (without DNA) was prepared and each of the 47 spruce DNA extracts was barcoded. In brief, each DNA extract (500 ng) was digested with restriction enzyme *Ape*KI for 2 hours. The details of oligonucleotide sequences for the *Ape*KI barcode adapters and temperature cycles are provided in Chen et al. [23]. Ligation products from each DNA extract were pooled and purified using QIAquick PCR purification kit (Qiagen). The amplified 48-plex libraries were diluted and sequenced (single-end reads only) twice on the Illumina HiSeq 2000 at the Cornell University Genomics Core Laboratory to achieve the sequencing coverage equivalent to 24-plex. Raw DNA short-read sequences were analyzed with a pipeline, the Universal Network Enabled Analysis Kit (UNEAK), tailored to species lacking reference genome information [27]. This SNP detection pipeline is available in TASSEL v5.0 [57]. To reduce sequencing error in genotype determination, we set the error tolerance rate to 0.03 (to pass the expected Illumina sequencing error rate at 0.4%). The resulting SNP table was further filtered using minimum value of inbreeding coefficient (mnF = 0.05) and minimum minor allele frequency (mnMAF = 0.05), and finally, SNPs that are present in less than 40% of the samples were eliminated from further analysis.

### Missing data imputation

To interpret missing values present in the filtered SNP set, five different imputation algorithms were employed: (1) mean imputation (MI), (2) singular value decomposition imputation (SVD:[28]), (3) traditional k nearest neighbor (kNN:[28]), (4) expectation maximization imputation (EM:[31]), and (5) k-nearest neighbor imputation but newly derived for half-sib family structure (kNN-Fam).

For SVD, the original SNP matrix was used to obtain a set of the *k* most significant eigenvectors of the SNP markers. The *k* eigenvectors were then used as predictors for linear regression estimation of the missing data. SVD was implemented in R [58] using the “bcv” pakage [59]. The resultant numerical SNP values (*x*) were further classified into three separate genotype classes, −1, 0, and 1. The classification algorithm was taken as a modified k-means algorithm [60], with the centroids set at −1 (*k*_*1*_), 0 (*k*_*2*_), and 1 (*k*_*3*_). The assignment of genotypes was done by satisfying:

1 $$ argmin\kern0.3em (SS)={\displaystyle \sum_{i=1}^k}{\displaystyle \sum_{x\in {S}_i}}x-{k}_i $$

where (1) defines the minimum distance for the SNP value from the centroids.

For traditional kNN, the missing values were replaced with the weighted average of SNP values at the k closest SNP markers. The distances between all possible pairs of markers were computed by Euclidean distance. We selected five families (6, 11, 17, 21, and 47) to test the imputation accuracy, as well as the efficiency of iterations for convergence (2, 3, 5 and 10 iterations for SVD; for EM, we tested the distance between the new estimate and the previous values less than 0.01). K = 10 and 30 were selected for accuracy estimates for kNN imputation. All iterations reached convergence criteria that were used in [61], however they resulted in different accuracies (shown in Additional file 4).

The kNN-Fam algorithm is derived from the kNN method of Troyanskaya et al. [28]. Missing values in the SNP table were first replaced with the mean of the locus by MI. A standardized genomic similarity matrix for all samples was calculated based on VanRaden [17] and the Euclidean distance between SNP markers was defined following Rutkoski et al. [61]. Instead of the classic k-nearest neighbor method, where

2 $$ \widehat{y}=\left(\frac{1}{K}\right) sigma(y) $$

the missing SNP values were replaced with:

3 $$ \widehat{y}= mode\left(\frac{1}{K1+K2}y\right) $$

where *K1* is the number of neighbors within the half-sib family based on the genomic similarity, *K2* is the number of neighbors from outside the family based on the Euclidean distance, and *y* is the original locus mean. We conducted exhaustive search for the optimal values of *K1* and *K2*, by permutating *K1* through 1 to 30 (the nearest neighbor set as 1, and then 2, 5, 10, 15, 20 to the maximum family size of 30), and *K2* from 1 to 250, as the total sample size of the panel is 1,126. The accuracy of kNN-Fam imputation was conducted for each permutation by randomly masking one million known data points from the filtered SNP table of the 5 selected families, and calculating the percentages of markers being imputed back to the correct SNP values.

### Phenotypic data

The studied trees were phenotyped for (a) two growth traits (height in m (HT) and diameter at breast height in cm (DBH) which were subsequently used to estimate stem volume in m^3^ (VOL) following Millman’s formula) (Millman M. Metric Volume and V-Bar Tables Derived from the British Columbia Forest Service Whole Stem Cubic Meter Volume Equations. Vancouver BC, 1976. Unpublished) and (b) three wood quality attributes (wood density in kg/m^3^ using X-ray densitometry (WD_X-ray_), resistance to drilling (WD_Res_), and acoustic velocity in km/s (V_Dir_)) [62]. Furthermore, WD_X-ray_ and V_Dir_ were used to derive the dynamic modulus of elasticity (MoE_d_) [63]. WD_X-ray_ is commonly used to estimate wood density using increment cores extracted from the sampled trees, while WD_Res_ and V_Dir_ represent indirect (i.e., non-invasive) methods that rely on wood density for either creating resistance during drilling or the speed of transmitting sound though the wood, respectively [62].

### Estimated breeding values (EBV)

The breeding value for each tree was estimated using ASReml v.3 using two different mixed linear models [64]. The first used the pooled populations to estimate multi-site breeding values (MSEBV), while the second was used to estimate single-site breeding values (SSEBV) as follows:

Multi-site model:

4 $$ y=\boldsymbol{X}\beta +{\boldsymbol{Z}}_1a+{\boldsymbol{Z}}_2s\beta +{\boldsymbol{Z}}_3sa+e $$

where *y* is the phenotypic measurement of the analyzed trait, *β* is a vector of fixed effect (i.e., the overall mean and the site effect), *a* is a vector of random additive effect of individual trees ~ N(0, ***A***σ^2^_a_), *sb* is a vector of the random effect of block within site ~ N(0, ***I***σ^2^_sb_), *sa* is a vector of random site x genotype interaction ~ N(0, ***I***σ^2^_sa_), *e* is a vector of random residual effect ~ N(0, ***I***σ^2^_e_), and ***X*** and ***Z***_*1*_-***Z***_*3*_ are incidence matrices assigning fixed and random effects to each observation and ***I*** and ***A*** are the identity and average numerator relationship matrices, respectively. Narrow-sense heritability was calculated as *h*^2^ = σ_a_^2^/(σ_a_^2^ + σ_sa_^2^ + σ_e_^2^) for the multi-site model.

Single-site model:

5 $$ y=\boldsymbol{X}\beta +{\boldsymbol{Z}}_1\beta +{\boldsymbol{Z}}_2a+e $$

This model is identical to the multi-site mixed linear model but without all terms related to site (site, block nested within site, and site x genotype interaction). Narrow-sense heritability was calculated as *h*^2^ = σ_a_^2^/(σ_a_^2^ + σ_e_^2^). Additionally, Genomic Best Linear Unbiased Predictor (GBLUP) [17] was used to estimate the narrow-sense heritabilities of the traits for single and multi-site using genotypes from imputed data produced by the EM algorithm with 30% missing data. This analysis was performed by substituting average numerator relationship matrix with marker-based relationship matrix [17] using observed allele frequencies.

### Genomic selection analyses

The SNP effects were estimated on the basis of two different methods: 1) Ridge Regression Best Linear Unbiased Predictor (RR-BLUP) implemented in R package rrBLUP [65] and 2) Generalized Ridge Regression (GRR) implemented in R package bigRR [38]. In both cases the following mixed linear models were fitted:

6 $$ y=\boldsymbol{X}\beta + \boldsymbol{Z}b+e $$

where *y* is the vector of EBV, *β* is the vector of fixed effect which is the overall mean, *b* is the vector of random SNP effects, ***X*** and ***Z*** are incidence matrices for *β* and *b*, respectively, ***X*** is a vector of 1 while ***Z*** was built from (-1, 0, 1) for aa, Aa and AA, respectively. The codes for ***Z*** were standardized according to the allele frequency using VanRaden’s method [17]. *β* and *b* are estimated simultaneously using Henderson’s mixed model equation (MME) [66]:

7 $$ \left(\begin{array}{cc}\hfill {\mathrm{X}}^{\hbox{'}}X\hfill & \hfill X\hbox{'}Z\hfill \\ {}\hfill {\mathrm{Z}}^{\hbox{'}}X\hfill & \hfill {\mathrm{Z}}^{\hbox{'}}Z+\lambda I\hfill \end{array}\right)\left(\begin{array}{c}\hfill \beta \hfill \\ {}\hfill b\hfill \end{array}\right)=\left(\begin{array}{c}\hfill {\mathrm{X}}^{\hbox{'}}y\hfill \\ {}\hfill Z\hbox{'}y\hfill \end{array}\right) $$

where $\lambda ={\widehat{\sigma}}_e^2/{\widehat{\sigma}}_b^2$ is the shrinkage parameter for the random SNP effects, so all the SNPs will have the same shrinkage magnitude, in other words, all are penalized to the same degree. In GRR, the SNPs with small effects are more penalized. The first step in GRR is an ordinary RR, then it again uses MME to fit the heteroscedastic model:

8 $$ \left(\begin{array}{cc}\hfill {\mathrm{X}}^{\hbox{'}}X\hfill & \hfill X\hbox{'}Z\hfill \\ {}\hfill {\mathrm{Z}}^{\hbox{'}}X\hfill & \hfill {\mathrm{Z}}^{\hbox{'}}Z+ diag\left(\lambda \right)\hfill \end{array}\right)\left(\begin{array}{c}\hfill \beta \hfill \\ {}\hfill b\hfill \end{array}\right)=\left(\begin{array}{c}\hfill {\mathrm{X}}^{\hbox{'}}y\hfill \\ {}\hfill Z\hbox{'}y\hfill \end{array}\right) $$

where diag ($\boldsymbol{\uplambda}$) is the diagonal matrix of SNP specific shrinkage parameters estimated as ${\lambda}_j={\widehat{\sigma}}_e^2/{\widehat{\sigma}}_{bj}^2$, where ${\widehat{\sigma}}_{bj}^2$ is variance attributed to j^th^ SNP and is estimated as:

9 $$ {\widehat{\sigma}}_{bj}^2 = \frac{{\widehat{b}}_j^2}{1-{h}_{jj}} $$

where, *b*_*j*_ is the SNP effect, and *h*_*jj*_ is the (*n* + *j*)^*th*^ diagonal element of the matrix ***H*** **=** ***T*** (***T***’***T***)^−1^***T’***, where

10 $$ T=\left(\begin{array}{cc}\hfill X\hfill & \hfill Z\hfill \\ {}\hfill 0\hfill & \hfill diag\left(\uplambda \right)\hfill \end{array}\right) $$

${\widehat{\sigma}}_{bj}^2$ is needed as it represents the form of implemented variable selection.

### Cross-validation, predictive accuracy and type-b genetic correlation

The predictive accuracy was estimated using a 10-fold cross-validation approach with 20 replications. In each replication, the data were randomly divided into 10 subsets (folds) and each one was used as validation population (representing 10% of the data set), while the remaining 9-folds were used as the training population (90% of the data set) to fit the GS model. This process was repeated 20 times with random assignment of the data to the 10 folds [67-69]. One advantage of this scheme is that it provides the degree of uncertainty (i.e., standard error) around these point estimates. In all the replicates, the models were fitted to the training data set and used to predict the GEBV of the validation data set by multiplying the vector of the marker effect estimated from the training population with the incidence matrix ***Z*** of the individuals in the validation population and summing over the estimated general mean:

11 $$ {\widehat{y}}_j=\widehat{u}+{\displaystyle \sum_i}{Z}_{ij}{\widehat{m}}_i $$

where *u* is intercept, ***Z*** is genotype at the i^th^ locus of the j^th^ individual and *m* is the marker effect. The accuracy of GS to predict the breeding value (BV) was estimated as the correlation of the vector of GEBV for all individuals (predicted from the validation step) with their estimated BV (MSEBV or SSEBV according to the validation scenario). As we used 20 replicates, we obtained 20 estimates for prediction accuracy and we estimated means and standard errors for these estimates. The developed models were validated under the following four scenarios, namely, (1) within site, (2) in all 6 possible combinations for cross-validation comparisons across sites, (3) as a multi-site population, where training and validation populations were derived from the combined population for cross-validation and (4) again as a multi-site population, but where the entire multi-site population was used as training population and the individual site as validation population.

Moreover, we estimated the type-b genetic correlation across sites, which is the additive genetic correlation between the traits measured on different individuals from the same genetic group but present in different environments, using a method described by Burdon [44].

### Multi-trait GS model

We applied Principle Component Analysis (PCA) to distil the correlated variables (EBV) into a set of linearly independent variables (i.e., the principal components (PCs)). We used HT, DBH, V_Dir_, WD_Res_, and WD_X-ray_ EBVs as variables to determine the PCs that best express these phenotypes and used their score as a new phenotype in subsequent RR-BLUP GS model for the multi-site scenario using the kNN-Fam imputation.

### ABLUP vs. GBLUP elite genotype selection comparison

Notwithstanding the relatively small number of 25 open-pollinated families under investigation, to illustrate the benefits of incorporating genomic information in selection, we conducted a selection exercise of 40 elite genotypes for inclusion into a hypothetical production population (seed orchard) following the group merit selection scheme of Lindgren and Mullin [70]. Group merit selection is founded on penalizing the average BV of a selected subset by increasing the weight on the entire group co-ancestry (measured by co-ancestry coefficient) to reach a desired “status number (*N*_*s*_)” [71] which is an approximation of the effective number of parents (*N*_*e*_) (i.e., measure of diversity). In this method, the co-ancestry coefficients are estimated from the pedigree values of the selected individuals (ABLUP) while in the GBLUP case, we used the marker-based relationship matrix [17] to approximate the co-ancestry of the selected individuals and their diversity was estimated by the number of founder genome equivalents (*N*_*ge*_: [72]).

### Study approval

Not required.

### Availability of supporting data

The data sets supporting the results of this article are available in the Dryad Digital Repository, http://doi.org/10.5061/dryad.8kb37.

## Additional files

Additional file 1:
**Imputation accuracy of kNN-Fam method with different K1 and K2 values.**
Additional file 2:
**GS prediction accuracies for GRR model for the seven studied traits for within single site, cross-sites, within multi-site, and for multi-site to single site with single- and multi-site (single- and multi-site GBLUP heritabilities are presented).**
Additional file 3:
**Cross-site GS prediction accuracies for all studied combinations (GS prediction model, imputation method, and trait).**
Additional file 4:
**The comparison of imputation methods.**
