# Supplementary material for: Prediction accuracies for growth and wood attributes of interior spruce in space using genotyping-by-sequencing
Source: BMC Genomics. 2015 May 9;16(1):370. doi: 10.1186/s12864-015-1597-y (PMC4424896; doi:10.1186/s12864-015-1597-y)
Supplement: Additional file 1: — Imputation accuracy of kNN-Fam method with different K1 and K2 values. [file 12864_2015_1597_MOESM1_ESM.docx]

Additional file 2.

Imputation accuracy of kNN-Fam method with different K1 and K2 values

| **K2: K value selected from non-family samples** | | | | | | | | | | |
| --- | --- | --- | --- | --- | --- | --- | --- | --- | --- | --- |
| **K1** | **1** | **2** | **3** | **4** | **5** | **10** | **20** | **50** | **100** | **250** |
| **1** | 0.771 | 0.818 | 0.819 | 0.830 | 0.832 | 0.843 | 0.848 | 0.848 | 0.846 | 0.845 |
| **2** | 0.816 | 0.816 | 0.830 | 0.830 | 0.837 | 0.845 | 0.848 | 0.848 | 0.846 | 0.845 |
| **5** | 0.824 | 0.834 | 0.835 | 0.839 | 0.840 | 0.846 | **0.849** | 0.848 | 0.846 | 0.845 |
| **10** | 0.837 | 0.838 | 0.842 | 0.842 | 0.843 | 0.845 | 0.848 | 0.848 | 0.846 | 0.845 |
| **15** | 0.840 | 0.842 | 0.843 | 0.843 | 0.844 | 0.846 | 0.848 | 0.848 | 0.846 | 0.845 |
| **20** | 0.840 | 0.842 | 0.843 | 0.843 | 0.844 | 0.845 | 0.848 | 0.848 | 0.846 | 0.845 |
| **30** | 0.842 | 0.843 | 0.844 | 0.844 | 0.844 | 0.846 | 0.847 | 0.847 | 0.846 | 0.845 |
