# Supplementary material for: Prediction accuracies for growth and wood attributes of interior spruce in space using genotyping-by-sequencing
Source: BMC Genomics. 2015 May 9;16(1):370. doi: 10.1186/s12864-015-1597-y (PMC4424896; doi:10.1186/s12864-015-1597-y)
Supplement: Additional file 2: — GS prediction accuracies for GRR model for the seven studied traits for within single site, cross-sites, within multi-site, and for multi-site to single site with single- and multi-site (single- and multi-site GBLUP heritabilities are presented). [file 12864_2015_1597_MOESM2_ESM.docx]

Additional file 3:

GS prediction accuracies for GRR model for the seven studied traits for within single site, cross-sites, within multi-site, and for multi-site to single site with single- and multi-site (single- and multi-site GBLUP heritabilities are presented).
