# Supplementary material for: Prediction accuracies for growth and wood attributes of interior spruce in space using genotyping-by-sequencing
Source: BMC Genomics. 2015 May 9;16(1):370. doi: 10.1186/s12864-015-1597-y (PMC4424896; doi:10.1186/s12864-015-1597-y)
Supplement: Additional file 4: — The comparison of imputation methods. [file 12864_2015_1597_MOESM4_ESM.docx]

Additional file 1

The comparison of imputation methods

|  | **MI** | **SVD_2** | **SVD_3** | **SVD_5** | **EM_0.01** | **EM_0.001** | **kNN_10** | **kNN_30** |
| --- | --- | --- | --- | --- | --- | --- | --- | --- |
| **family 11** | 0.799 | 0.806 | 0.805 | 0.800 | 0.771 | 0.771 | 0.799 | 0.795 |
| **family 17** | 0.808 | 0.812 | 0.810 | 0.803 | 0.778 | 0.777 | 0.788 | 0.809 |
| **family 21** | 0.805 | 0.805 | 0.801 | 0.795 | 0.769 | 0.768 | 0.769 | 0.800 |
| **family 6** | 0.800 | 0.822 | 0.809 | 0.802 | 0.767 | 0.767 | 0.799 | 0.802 |
| **family 47** | 0.803 | 0.802 | 0.800 | 0.798 | 0.770 | 0.770 | 0.773 | 0.809 |
| **Average** | 0.803 | 0.810 | 0.805 | 0.799 | 0.771 | 0.771 | 0.785 | 0.803 |
|  |  |  |  |  |  |  |  |  |
